# Current insights and assumptions on α-synuclein in Lewy body disease

**DOI:** 10.1007/s00401-024-02781-3

**Published:** 2024-08-14

**Authors:** Rehana K. Leak, Rachel N. Clark, Muslim Abbas, Fei Xu, Jeffrey L. Brodsky, Jun Chen, Xiaoming Hu, Kelvin C. Luk

**Affiliations:** 1 Graduate School of Pharmaceutical Sciences, Duquesne University, 418C Mellon Hall, 913 Bluff Street, Pittsburgh, PA 15219 USA; 2 Department of Neurology, University of Pittsburgh, Pittsburgh, PA USA; 3 Department of Biological Sciences, University of Pittsburgh, Pittsburgh, PA USA; 4 Department of Pathology and Laboratory Medicine, University of Pennsylvania, Pennsylvania, PA USA; 5 Geriatric Research, Education and Clinical Center, Veterans Affairs Pittsburgh Health Care System, Pittsburgh, Pennsylvania USA

**Keywords:** Synuclein, Lewy body, Neurodegeneration, Parkinson’s disease, Prion, Dementia

## Abstract

Lewy body disorders are heterogeneous neurological conditions defined by intracellular inclusions composed of misshapen α-synuclein protein aggregates. Although α-synuclein aggregates are only one component of inclusions and not strictly coupled to neurodegeneration, evidence suggests they seed the propagation of Lewy pathology within and across cells. Genetic mutations, genomic multiplications, and sequence polymorphisms of the gene encoding α-synuclein are also causally linked to Lewy body disease. In nonfamilial cases of Lewy body disease, the disease trigger remains unidentified but may range from industrial/agricultural toxicants and natural sources of poisons to microbial pathogens. Perhaps due to these peripheral exposures, Lewy inclusions appear at early disease stages in brain regions connected with cranial nerves I and X, which interface with inhaled and ingested environmental elements in the nasal or gastrointestinal cavities. Irrespective of its identity, a stealthy disease trigger most likely shifts soluble α-synuclein (directly or indirectly) into insoluble, cross-β-sheet aggregates. Indeed, β-sheet-rich self-replicating α-synuclein multimers reside in patient plasma, cerebrospinal fluid, and other tissues, and can be subjected to α-synuclein seed amplification assays. Thus, clinicians should be able to capitalize on α-synuclein seed amplification assays to stratify patients into potential responders *versus* non-responders in future clinical trials of α-synuclein targeted therapies. Here, we briefly review the current understanding of α-synuclein in Lewy body disease and speculate on pathophysiological processes underlying the potential transmission of α-synucleinopathy across the neuraxis.

## Introduction

Lewy body disorders are multisystemic conditions with clinical symptoms spanning from mild to severe deficits in olfaction, peripheral autonomic and enteric function, mood/affect, sleep/wake cycles, movement, memory, and/or cognition. Lewy body disorders include, but are not limited to, Parkinson’s disease (PD), Parkinson’s disease dementia (PDD), dementia with Lewy bodies (DLB), incidental Lewy body disease (ILBD), and primary autonomic failure (PAF). Lewy body disorders are histologically categorized by abnormal inclusions known as Lewy bodies in neuronal somata and Lewy neurites within neuronal processes. Lewy pathology is not uncommon at autopsy. Although estimates vary, ~ 8 to 17% of subjects sixty years or older display ILBD [25, 60, 81, 122], and Lewy pathology is present in ~ 40 to 57% of Alzheimer’s patients [51, 94, 136, 182, 249]. Lewy bodies may also be observed in some cases with Type I Gaucher disease and parkinsonism or in neurodegeneration with brain iron accumulation type 1 [6, 173, 273].

## α-Synuclein is a key—Albeit partial—component of Lewy bodies

In this review, we will focus on a critical component of Lewy inclusions, the protein α-synuclein. Lewy bodies are partly composed of abnormal, filamentous aggregates of α-synuclein [110, 232, 233]. The objective of this review is to encourage speculation on mechanisms underlying the potential transmission of α-synuclein pathology across the central nervous system. This is an important topic because the molecular components of Lewy bodies, their morphology, and the spatiotemporal patterns of inclusion formation and neurodegeneration may dictate the type of Lewy body disorder that the patient develops. Although neurodegeneration is a key driver of neurological deficiencies, this does not rule out the possibility that living, α-synuclein aggregate-bearing neurons are functionally compromised in patients with Lewy body disease and contribute to maladaptive neurological traits. On the other hand, the region-specific pattern of α-synuclein aggregation does not strictly parallel regional patterns of cell death [87, 241]. Similarly, if all neurons housing Lewy inclusions were sufficiently dysfunctional, one would expect a robust positive correlation between Lewy-related pathologies and neurodegeneration or movement deficits, but this is not universally observed. Although Lewy-related pathology across the brain was shown to be correlated with neuronal loss in the substantia nigra and with motor deficits in cases without concomitant Alzheimer’s disease [14], other studies report a lack of correlation of nigral neurodegeneration with Lewy body distribution or density [183]. A third study demonstrated a negative association between nigral neuronal densities and α-synuclein^+^ profiles [61], but the latter report also showed that the demise of nigral neurons and loss of dopaminergic markers can *precede* the emergence of nigral α-synuclein aggregates, confirming earlier work [61, 164]. Some brain regions, such as the supraoptic and paraventricular hypothalamic nuclei, and selective parts of the hippocampal formation and presupplementary motor cortex may also display neuronal loss with minimal Lewy-related pathologies [4, 20, 150, 194]. Hence, despite being the namesake, Lewy bodies are not the sole source of pathology in Lewy body disease. Among other possibilities, the potential uncoupling of cell death and α-synuclein aggregation may reflect a protective nature of some of the diverse types of Lewy bodies, at least within neurons that are already diseased, as speculated below.

**Fig. 1:**
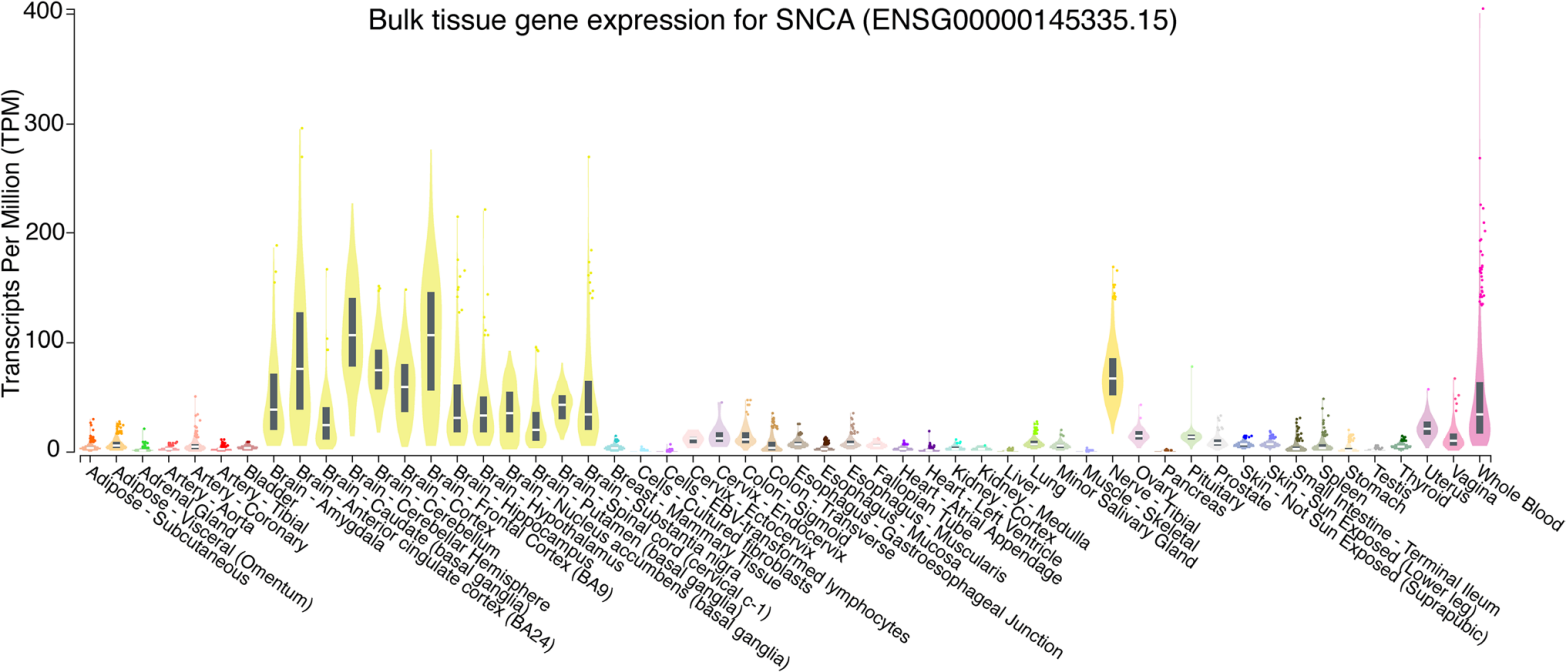
Bulk tissue expression of *SNCA* according the GTEx online data portal hosted by the Broad Institute of MIT and Harvard. The Y axis is shown on a linear scale and the X axis is sorted alphabetically according to tissue type or brain region. Note the high median expression of *SNCA* within human cerebellar tissues, discussed below alongside Fig. 2. These violin plots were retrieved from the GTEx portal on July 27, 2024 at this link: https://www.gtexportal.org/home/gene/SNCA

α-Synuclein is encoded by the phylogenetically conserved *SNCA* gene. *SNCA* expression is normally expressed in presynaptic terminals of the vertebrate nervous system, but it is also highly enriched in other tissues, such as blood (Fig. 1). As with many genes, *SNCA* is pleiotropic, and α-synuclein plays subtle roles in an array of synaptic functions, including neuroplasticity, neurotransmitter release, SNARE complex assembly, vesicle trafficking and clustering, fusion pore formation, endocytosis, tyrosine hydroxylase activation, and dopamine synthesis and transport [36, 37, 44, 79, 132, 190, 217, 220, 221, 239, 245, 266, 269]. Although *Snca* gene deletion in mice does not elicit dramatic changes in neurotransmission, neuronal viability, and/or behavioral outcomes (even when combined with deletions of other members of the synuclein family), laboratory mice are typically evaluated when young, whereas the impact of loss of α-synuclein is more evident in old animals [5, 89, 174]. Thus, when inclusions materialize in Lewy body disease, the manifestation of neurological symptoms may depend on age-dependent loss of normal α-synuclein functions in the synapse and other subcellular locations. Aside from this loss-of-function phenotype, there may also be a toxic gain of function of the abnormal α-synuclein aggregates, including interference with axonal transport [257, 259, 274] and clogging of protein degradation systems [53, 154, 235, 256]. The gain-of-function scenario does not exclude the possibility that the growth and maturation of Lewy bodies also benefits the diseased neuron [69]. For example, the formation of Lewy bodies may lower the concentration of soluble α-synuclein oligomers with more harmful properties than mature α-synuclein filaments. Lewy bodies may thus serve to sequester damaged cellular components that escape or saturate degradation systems (e.g., lysosomal and proteasomal digestion). In other words, there may be both advantages and disadvantages to formation of inclusions within diseased neurons, and the *net* outcome of these diverse responses may be the final arbiter of cell fate.

The gene *SNCA* lies along the long arm of chromosome 4q21-q23, and point mutations (e.g., V15A, A30P, E46K, H50Q, G51D, A53T, and A53V) or wildtype gene duplications and triplications have been identified in familial cases of Lewy body disease [23, 24, 45, 67, 74, 104, 106, 126, 191, 226, 277]. The number of *SNCA* alleles is dose-dependently linked to Lewy body disease severity, with cases of duplication causing milder forms of late-onset PD, whereas triplications cause severe clinical manifestations, with parkinsonism, autonomic dysfunction, dementia and, in some cases, vacuolation of cortical tissue [71, 107, 170, 203, 227]. Although more than 90% of PD cases do not adhere to Mendelian patterns of inheritance, all cases of Lewy body disease likely have a genetic component that intersects with disease triggers in the environment [227]. Numerous risk loci for PD exist, but genome-wide association studies consistently demonstrate that specific sequence variations in *SNCA* are risk factors for Lewy body disease (and an earlier age of disease onset), particularly when the variants raise *SNCA* expression levels [23, 24, 57, 206, 223]. In cases with *SNCA* triplication, a 1.8-fold increase in levels of soluble α-synuclein protein was observed in blood samples, along with a doubling of *SNCA* mRNA in brain tissues, with corresponding increases in insoluble α-synuclein species of high molecular mass [165], suggestive of aggregate formation.

Emerging experimental evidence suggests that the backbones of the inclusion filaments, the abnormal α-synuclein aggregates, are transmitted from cell to cell across interconnected brain regions and serve as nucleation sites for additional Lewy inclusion formation [84, 86, 189, 250]. Thus, aggregated forms of α-synuclein stoke the fibrillization of neighboring α-synuclein monomers into β-sheet-rich fibrils typical of amyloid material [85, 212, 218]. Although intense α-synuclein immunoreactivity is observed within Lewy bodies and Lewy neurites [75, 128, 232, 233], it is not clear if α-synuclein is truly the most abundant component by mass [219]. Indeed, Lewy inclusions contain numerous other proteins, including those related to PD risk, such as DJ1, parkin, and PINK1. The inclusions house proteins involved in proteasomal and autophagic degradation systems (e.g., ubiquitin, ubiquitin C-terminal hydrolase, proteasome activators 28 and 700, and autophagic adaptor p62/sequestosome-1), proteins associated with aggresomes (pericentrin, γ-tubulin, and HDAC6), as well as microtubule-associated proteins, heat shock proteins, and kinases [135, 140, 263]. Lewy bodies also harbor lipids and fragments of lysosomes, mitochondria, proteasomes, and the cytoskeleton [168, 219, 263]. Thus, the inclusions are remarkably heterogeneous, as may be reflected in their potential cytotoxic versus cytoprotective roles, and in their diverse histological staining properties. Lewy bodies can be eosinophilic, weakly eosinophilic (pale bodies), or hematoxylin and eosin-negative and they can react with thioflavin dyes and lipid-binding agents [86, 168]. Lewy bodies are also found at varying stages of compaction and maturity in different neurons; some Lewy bodies may develop a dense core and peripheral halo where α-synuclein immunoreactivity is concentrated, whereas others may harbor α-synuclein immunoreactivity without a core and shell [263].

## Properties of α-synuclein in the context of Lewy body disease

α-Synuclein is widely recognized as a soluble protein, but it likely exists in equilibrium between soluble cytosolic forms and phospholipid-bound forms along membranous structures, where it affects their curvature [43, 162, 255]. An 11-residue sequence (XKTKEGVXXXX) repeated seven times in the 140 amino acid sequence of α-synuclein facilitates the formation of an amphipathic α-helical fold, which associates with phospholipid membranes and displays preference for smaller vesicles, such as ~ 40 nm synaptic vesicles [37]. The *SNCA* missense mutations associated with Lewy body disease tend to code for amino acids within these eleven-residue repeats. Residues 1 to 60 along the amino terminus of α-synuclein house the membrane-binding motifs important for formation of the α-helices. The central non-β-amyloid component (NAC) region along residues 61–95 is hydrophobic and crucial for β-sheet formation, and the unstructured, negatively charged acidic and glutamate-harboring carboxyl terminus is subject to numerous post-translational modifications that may affect α-synuclein hydrophobicity and its interactions with proteins and lipids [37, 234].

α-Synuclein was commonly reported as intrinsically disordered, based on biochemical assays, but in human brain tissue subjected to protein cross-linking methods, approximately 75% of α-synuclein normally adopts an α-helical multimeric form [58]. The latter multimeric form is not pathologic and plays a physiologic role in synaptic vesicle clustering [266] but it is destabilized and less abundant in the presence of Lewy body disease-causing missense mutations [58, 59]. Thus, low physiologic α-synuclein multimer/monomer ratios may contribute to disease progression, as suggested by loss of dopaminergic neurons and emergence of L-DOPA responsive motor symptoms in mice forced to express multiple E → K mutations in the KTKEGV motifs or similar multiplications of the G51D mutation in α-synuclein [176, 177].

As noted above, α-synuclein is subject to post-translational modifications that include, but may not be limited to, ubiquitination at lysine residues, phosphorylation, oxidation, acetylation, O-GlcNAcylation (e.g., [12]), sumoylation, nitration (e.g., [35]), and N- or C-terminal truncation (e.g., [141]). Spillantini and colleagues originally observed that antibodies raised against ubiquitin tend to label fewer Lewy inclusions than antibodies against α-synuclein [232, 233], consistent with the notion that ubiquitination of α-synuclein occurs subsequent to deposition in Lewy inclusions [3, 83]. This may also be true of phosphorylation events, but the probability of a post-translational modification will naturally rise with the age of the protein and with impediments to protein turnover/clearance, as will the probability that the protein denatures and precipitates into an insoluble inclusion.

Candidate kinases playing a role in α-synuclein phosphorylation include casein kinase I and II, G-protein-coupled receptors 1, 2, 5, and 6, and polo-like kinases [37, 39, 118]. The phosphorylation sites along α-synuclein include serine 87 or 129, and tyrosine 125, 133, or 136 [37], but the hyperphosphorylation of serine 129 is perhaps the most useful modification in distinguishing Lewy body disease. In brain tissues from patients with Lewy body disease, nearly the entire fraction of aggregated α-synuclein is phosphorylated at serine 129, whereas a low percentage of the soluble α-synuclein fraction is phosphorylated at this residue in control and DLB brains [3, 75, 178, 181]. The latter, physiological phosphorylation of synaptic α-synuclein at serine 129 is linked with neuronal activity and facilitates the association of α-synuclein with other synaptic proteins, such as VAMP2 and synapsin [185, 197]. There are numerous conflicting reports of the causal impact of phosphorylation of α-synuclein on neurotoxicity and Lewy inclusion formation (e.g., [10, 46, 72, 88, 159, 270]), but recent work suggests that phosphatases are unable to effectively dephosphorylate α-synuclein molecules that are sheltered within inclusions [49] and that the hyperphosphorylation event may serve to prevent further aggregation of α-synuclein [80].

Although there is utility in employing pSer129 antibodies to identify Lewy-related pathologies, the use of pSer129 antibodies to identify Lewy bodies and Lewy neurites is associated with technical artifacts, as is common with phospho-specific antibodies (see [2]). Therefore, in situ or biochemical confirmation of Lewy or Lewy-like inclusions is recommended via colabelling with various antibodies that react with Lewy inclusions (e.g., multiple antibodies against α-synuclein, ubiquitin, and/or p62), use of electron microscopy and amyloid stains (e.g., Thioflavin derivatives), and leveraging α-synuclein insolubility in nonionic detergents, resistance to proteinase K, and proteolytic truncation of its carboxyl terminus (e.g., [141]).

*SNCA* is highly expressed in the central nervous system, but its mRNA is also present in peripheral tissues, such as blood, uterine, ovary, prostrate, colon, esophageal, and skin tissues, with highest bulk expression in cerebellar tissues and lowest expression within skeletal muscle (Fig. 1). Some peripheral tissues may release α-synuclein protein into the interstitial fluid and plasma, but the source of transmissible α-synuclein aggregates in Lewy body disease is likely to originate in neurons, given *SNCA* expression patterns at the tissue level in Fig. 1 and enrichment in synaptic terminals at the cellular level.

Aside from α-synuclein expression in diverse tissue types, the intracellular heterogeneity of Lewy inclusions was originally recorded by their discoverer, Fritz Lewy, who drew various shapes of inclusions along cellular processes, within neuronal somata, and inside cell nuclei (see Fig. 3 of [86]). Since then, α-synuclein has been reported in nonsynaptic compartments such as the Golgi apparatus and nucleus [19, 125, 209, 271]. Indeed, the term α-synuclein was coined as a portmanteau of the words synapse and nucleus, as it was reported to lie along the inner lining of the neuronal nuclear envelope, aside from being concentrated in synaptic terminals [156]. Controversies surrounding the histological work [240] could reflect lack of specificity of the older antibodies or higher levels of α-synuclein in the synaptic terminal compared to nucleus.

Given the broad tissue distribution of α-synuclein and breadth of neurological symptoms in Lewy body disorders, it is unsurprising that Lewy-related aggregates form not only in the brain and spinal cord, but also in the skin [82, 96], submandibular gland [16, 179], autonomic ganglia [15], heart [113, 179], adrenal medulla [76], and gastrointestinal plexuses of Meissner and Auerbach [193, 262]. Although the anatomical reach of Lewy pathologies may not be as spatially expansive as the normal expression of *SNCA*, the latter may also have diagnostic significance.

## α-Synuclein aggregation is prompted by a stealthy disease trigger

Braak and colleagues originally speculated that Lewy pathology is initiated by exposure of cranial nerve endings in the gastrointestinal and nasal mucosal linings [31–33, 98, 99]. Thus, once cranial nerves I and X are exposed to the causative agent and aggregates of α-synuclein take shape, the aggregates were proposed to spread from cell to cell via neuroanatomical fiber tracts, perhaps explaining olfactory and autonomic symptoms at the earliest stages of PD [29–33, 56]. Prior to this unconventional notion, PD had often been described as a motor condition with confined, selective degeneration of the substantia nigra in the ventrolateral mesencephalon and perhaps the locus coeruleus of the pons (another catecholaminergic structure housing melanin pigment). Historical biases towards catecholamine-releasing neurons and melanin-rich brain regions understandably reflect the evolution of histochemical and microscopic techniques in the last century. In addition, animal studies also supported the view that selective loss of nigrostriatal fibers underlies the motor deficits of PD [279], while clinical evidence confirmed the utility of L-DOPA to help replace dopamine stores.

The idea that attractions between proteins culminate in their ordered assembly into filaments that are deposited in the intra- or extracellular space also arose decades ago (e.g*.*, [114, 214, 247, 264, 265]. The molecular conversion event of α-synuclein monomers into filaments that precipitate and help form Lewy inclusions is akin to the prion diseases (e.g., Kuru and fatal familial insomnia) [38, 231, 268]. Although α-synucleinopathies are not (yet) viewed as infectious conditions with inter-organismal transmission in the wild (see [70, 130]), human fetal brain cells transplanted into the brains of patients with PD eventually develop Lewy bodies [124, 138, 139]. The latter findings strongly support human cell-to-cell transmission of Lewy-related pathologies but may also reflect a response to the diseased microenvironment of the host, leading to structural modifications and precipitation of otherwise normal α-synuclein molecules within grafted cells.

Although inclusions harboring α-synuclein filaments are the main hallmark of Lewy body disease, the aggregation of α-synuclein may be secondary to exposure to the disease trigger, even in inherited cases. Unless there is stochastic misfolding of α-synuclein, some other stimulus, such as environmental factors or *SNCA* overexpression/mutation, likely initiates the first aggregation event. During disease initiation, an environmental trigger may breach not only epithelial barriers within nasal, oral, esophageal, or gastrointestinal mucosa, but perhaps also the epidermis of the skin. One can further speculate that, although everyone is exposed to the disease trigger—including the spouses and coworkers of those with Lewy body disease—those with greater genetic predispositions (e.g., old age, male sex, defects in protein quality control pathways, inferior immune function) are more likely to develop a clinical syndrome. In addition, individuals could be exposed to the same disease trigger but at varying frequencies, durations, and concentrations (amplitude), as well as at different circadian phases. The temporal qualities and intensity of these environmental factors, when combined with compromised genetic defenses, conceivably influence the probability of α-synuclein aggregation. An alternative scenario is that only few individuals are exposed to the disease trigger, and only they will develop Lewy body disease.

The disease trigger(s) for idiopathic Lewy body disease has not been confirmed, but the inhalation and swallowing of (or perhaps skin contact with) toxicants such as trichloroethylene, paraquat, or natural toxins such as rotenone are possible culprits [54, 55, 172, 242], as are head trauma and dairy product ingestion [8, 196]. By suppressing energetic resources, eliciting oxidative stress, and disabling protein quality control mechanisms in parts of the neuraxis that contact peripheral boundaries, exposures to environmental vectors conceivably shift the shapes of α-synuclein molecules towards the pathologic forms. Based on emerging evidence, one can also speculate that environmental disease triggers encourage the first α-synuclein nucleation event by promoting liquid–liquid phase separation prior to droplet formation, followed by potential conversion into a perinuclear aggresome, and then maturation into a relatively insoluble inclusion [168, 171, 198, 199]. Host factors, such as polymorphisms in the amino acid sequence of α-synuclein could influence cellular thermodynamics in this scenario [152], particularly when genetically determined defenses are naturally low, cells are exposed to chronic environmental stressors, and *SNCA* transcription rates are higher in select neuron populations [91], leading to possible molecular crowding and phase changes of α-synuclein.

Aside from toxicant and toxin exposures, head trauma, and dairy product ingestion as potential disease triggers, a fourth possibility is that infections with microbial pathogens set the stage for immune cell dysregulation and α-synucleinopathy in peripheral and central neurons [97, 131, 137, 142, 188]. Recent experimental evidence suggests that, (1) α-synuclein plays a role in immunity and inflammation, (2) microbial gut amyloids stimulate α-synuclein aggregation, and (3) α-synuclein harbors antimicrobial properties under conditions of viral or bacterial infection after nasal or systemic inoculation [1, 17, 117, 204, 236, 243, 244, 246]. The evidence for inflammation and immune cell dysregulation in human Lewy body disease is partly based on imaging of translocator protein 18kDa (TSPO) (reviewed in [143, 213, 243, 244, 276]), but it has been difficult to ascertain if the *net* impact of immune cell function in Lewy body disease is helpful or harmful (it may also vary across genetically diverse human subjects).

Innate immune cells are the first line of defense within injured and diseased tissues, and in the immunospecialized CNS, this essential role is primarily executed by microglia/macrophages [77]. Neurodegenerative disorders are believed to be accompanied by microgliosis (i.e., recruitment, hyperplasia, and stimulation of microglia) and the hypersecretion of proinflammatory cytokines, chemokines, eicosanoids, free radicals, and proteases. However, microglia/macrophages are essential for CNS repair and recovery, particularly under conditions of injury and/or infection. Postmortem studies reveal numerous microglia-like cells immunopositive for the antigen-presenting cell marker, major histocompatibility complex class II antigen (HLA-DR) in nigral tissues of subjects with PD [160] and in the transentorhinal cortex of subjects with DLB [151], compared to control subjects. In addition, the area occupied by the microglia/macrophage lysosome marker CD68 (i.e., macrosialin) is higher in DLB brain tissues [237]. However, these effects were not observed for the pan-microglia/macrophage marker Iba1, which may indicate glial cell senescence, with no enhanced proliferation or reactivity [237]. Indeed, when microglia/macrophages are stimulated and turn reactive, they tend to hypertrophy, but this stress response is also not observed in brains with DLB [237]. On the other hand, higher levels of dystrophic (beaded/fragmented) microglia/macrophage processes are evident in DLB [11] and mRNA levels for the cytokine gene *Il-6* are higher in the hippocampi of subjects with PD and DLB [109]. The evidence for immune cell dysregulation in Lewy body disease reveals, at the least, a rich pleomorphism of microglia/macrophages [11].

## α-Synucleinopathy may be transmitted across the neuraxis

Following the seeding of α-synuclein aggregates in the rostral forebrain and caudal brainstem, Lewy pathology may be transmitted deeper into the brain, eventually affecting the locus coeruleus of the pons, the raphe complex, the pedunculopontine tegmentum, the substantia nigra and ventral tegmental area of the mesencephalon, the magnocellular basal forebrain nuclei, the amygdala and other limbic regions of the telencephalon, such as the entorhinal cortex and hippocampal subfield CA2 [29–33, 56]. Lewy pathology was originally hypothesized by Braak et al*.* to emerge within neocortical regions at final stages of PD, perhaps spreading to these destinations from meso- and allocortical sites, with the heavily myelinated primary motor and sensory cortices as the last to be affected [31, 32, 34].

The clinical staging of Lewy body disorders continues to be refined since Braak criteria were originally proposed. In 2009, Beach et al. reported a Unified Staging System, in which DLB and Alzheimer’s disease with Lewy bodies (ADLB) were found to be associated with limbic-predominant Lewy pathology in early stages of disease, whereas early PD was linked to brainstem-predominant pathology, with both types of disease breaching the neocortex at end stages [14]. Importantly, Beach and colleagues also suggested that the first brain region likely to be affected is the olfactory bulb rather than medulla oblongata [13, 14]. The staging of Lewy pathology by other comprehensive histological systems, such as that of McKeith or Leverenz et al*.*, and more recently, the Lewy pathology consensus criteria by Attems et al*.*, supports parcellation into olfactory-only, amygdala-predominant, limbic, brainstem, and neocortical stages [9, 136, 161]. Borghammer’s Brain-First vs. Body-First staging scheme also distinguishes cases with Lewy pathology in the autonomic nervous system or lower brainstem—which rarely display Lewy pathology in olfactory nuclei—from cases with amygdala-predominant Lewy pathology, which nearly always have Lewy pathology in olfactory nuclei [26–28]. Chahine and colleagues recently offered a biologic staging system for PD that also accounts for the presence of prodromal disease and proposes α-synuclein aggregation as a major risk factor for dopaminergic dysfunction [41, 225].

Although retrograde and anterograde transport of α-synuclein both contribute to intracellular expansion of aggregates, retrograde transport to the somata appears to predominate [101, 210]. Thus, neuritic forms of Lewy inclusions tend to appear before somal Lewy bodies, including the Lewy-like inclusions in experimental models (e.g., see [62]). Indeed, α-synuclein aggregation has been reported to commence in axon collaterals, supporting retrograde transfer to the cell body prior to formation of somal inclusions [116]. Retrograde degeneration of neurons also seems more likely, given that synaptic and axonal deficits in Lewy body disease also precede loss of neuronal somata. Thus, there is greater fractional loss of dopaminergic terminals in the striatum compared to degeneration of nigral neurons in PD [47, 78, 274].

In both rodents and nonhuman primates, infusions of preformed α-synuclein fibrils into dorsal striata precipitate the (retrograde) onset of Lewy-like inclusions in nigral efferent neurons and other (anterogradely and retrogradely) anatomically connected structures [50, 144–148, 187, 201, 210, 251]. Nigral cell loss, deficiencies of dopaminergic tone in the striata, and L-DOPA-responsive behavior deficits are thereby induced, suggesting that striatal infusions of α-synuclein fibrils model mid-to-late disease, according to Braak staging criteria. Infusions of oligomeric forms tend to be less successful in recapitulating disease [73].

As Lewy pathologies may emerge earlier in the olfactory bulb than in nigral efferents, the olfactory bulb has also been infused with preformed fibrils in mice, rats, and monkeys to model Lewy body disease [21, 22, 49, 50, 157, 158, 166, 167, 201, 202, 208]. As expected, dense Lewy-like pathologies emerge in limbic regions of the telencephalon connected to the olfactory site of infusion, such as the cortical amygdala, piriform (primary olfactory) and entorhinal cortices, and the ventral hippocampal formation (first sector of Ammon’s horn). The inclusion patterns appear like a moonlike crescent along lateral, ventral, and dorsolateral boundaries of the telencephalon, as is readily appreciated along sagittal or horizontal planes of section. A limitation of this type of work is that naturally formed, human wildtype fibrils differ structurally from fibrils synthesized in vitro from rodent *Snca*, in ways that may alter templating and propagation properties, as well as the kinetics of cell-to-cell transfer [251, 278]. It should be acknowledged that animals do not develop the Lewy bodies that are found in human brains, and that it is difficult to faithfully recapitulate Lewy body disease in the lab. The attributes of α-synucleinopathy that are described in short-lived rodents may therefore not translate to age-related human conditions. Another limitation of the animal models is that rodents are macrosmatic creatures with possibly stronger, more active connections in brain regions involved in olfaction, compared to microsmatic (visual) primates. As discussed below, the density of synaptic terminals and synaptic activity levels (including pinocytic rate during vesicular recycling) may influence the probability of developing Lewy-related pathologies.

In cynomolgus monkeys, preformed fibril infusions in the striata elicit mild loss of nigral neurons, with a parallel rise in the dopaminergic transporter (DAT) [50]. Increases in DAT and tyrosine hydroxylase are also observed after fibril infusions in the bulbar anterior olfactory nucleus (Fig. 11 in [158]). These observations in mice and nonhuman primates may be analogous to the *Parkinson's Progression Marker Initiative* (PPMI) showing higher DAT in as yet-unaffected mutant *GBA* carriers—prior to the onset of clinical parkinsonism [224]. However, in most studies with long survival periods after infusions of fibrils into the striatum, dopaminergic markers are lowered, and mild to moderate loss of nigral neurons is observed [63, 147, 186, 187, 252]. It is still unclear whether patients who are exposed to a disease trigger but have not developed ILBD display compensatory increases in enzymes that lie along the biosynthetic pathway for dopamine and/or show compensatory sprouting of dopaminergic terminals in the striatum. These types of adaptive changes could be manifested as increases in tyrosine hydroxylase and DAT markers, at least in the earliest stages of disease.

## Current assumptions on Lewy body disease

**Fig. 2:**
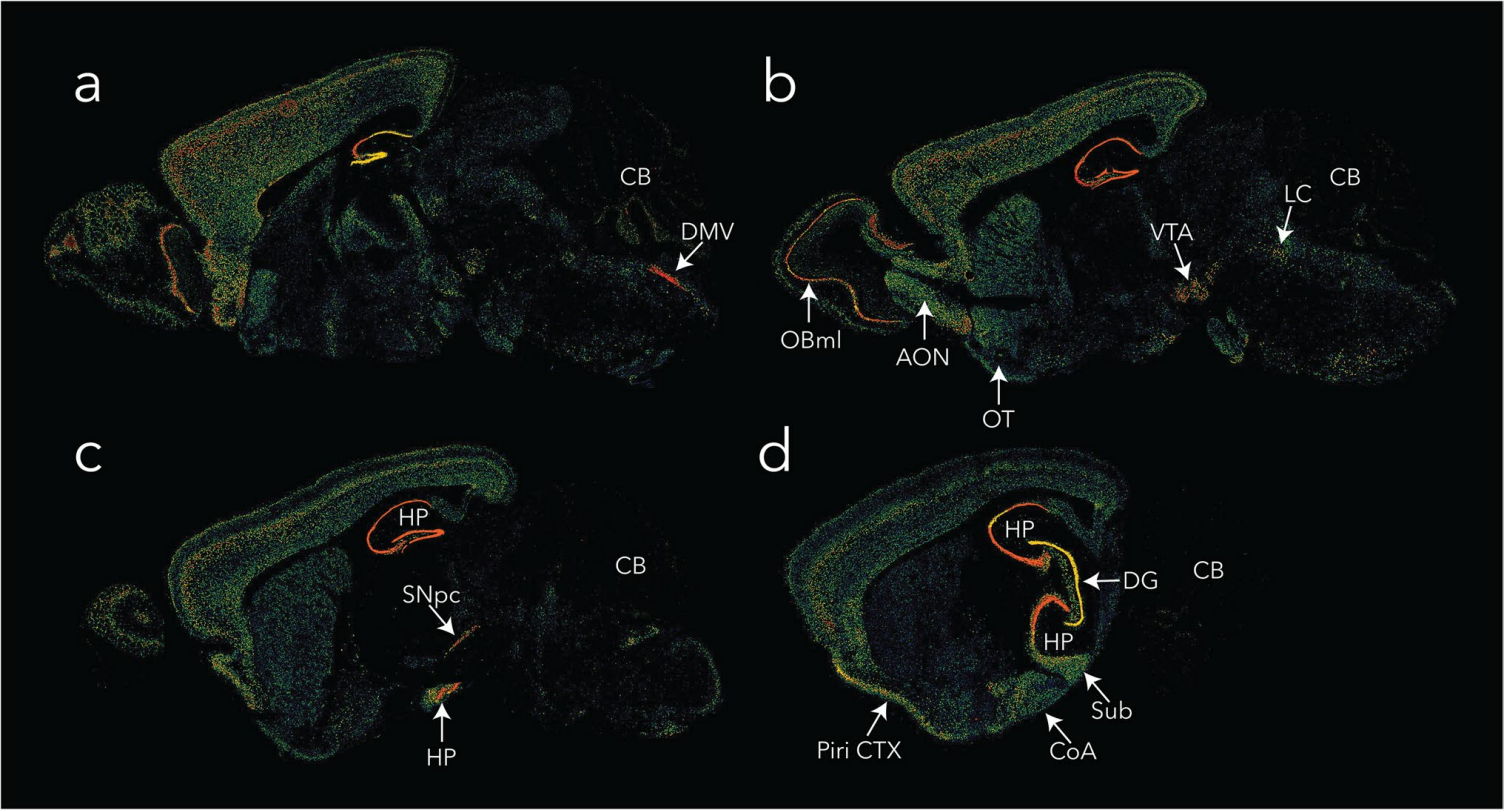
Allen Brain Atlas in situ mRNA hybridization for *Snca*, the mouse gene encoding α-synuclein [134]. **A**–**D** Sagittal brain sections from a 56-day-old C57BL/6 J male mouse are shown from medial to lateral, with high expression of α-synuclein mRNA in the dorsomotor nucleus of the vagus (DMV), the substantia nigra pars compacta (SNpc), the pyramidal and granule cells of the hippocampus (HP), and the mitral cell layer of the olfactory bulb (OBml). Moderate expression is seen in the ventral tegmental area (VTA), anterior olfactory nucleus (AON), the locus coeruleus (LC), the subiculum (Sub), the cortical amygdala (CoA), the piriform cortex (Piri CTX). There is low expression of *Snca* in the olfactory tubercle (OT), cerebellum (CB), and white matter (unlabeled). Antisense data were retrieved from the Allen Institute at this link: https://mouse.brain-map.org/experiment/siv?id=79904550&imageId=79764405&initImage=expression&contrast=0.5,0.5,0,255,4
  The cerebellum does not routinely develop Lewy pathologies [68] and has overall low *Snca* expression in mice (Figure 2) but unexpectedly high *SNCA* expression in humans (Figure 1), suggestive of species differences. On the other hand, mRNA levels may not scale in proportion to protein expression levels. Thus, the human cerebellum displays low expression of pan-α-synuclein protein [68] despite relatively high *SNCA* mRNA (Figure 1).
  It is notable that *Snca* is expressed at low levels in the mouse cerebellum of Fig. 2 while α-synuclein protein levels are relatively high in rat cerebellar tissues (see Fig. 2a in [112]). However, Fig. 3 shows single-cell RNA sequencing data from the DropViz database, and lists mouse cerebellar interneurons as harboring high *Snca* expression [207]. In this respect, it is worth noting that the cerebellum also contains a high proportion of white matter, and myelinated neurons do not typically develop Lewy pathology [31, 32, 34]. Thus, some cellular subtypes within the cerebellum will have more α-synuclein expression than others, potentially leading to variance across studies that rely on different methods and have varying degrees of cellular resolution.

With the intention of accelerating research on Lewy body disease, we will list some of the additional factors known or speculated to influence α-synucleinopathy:

1. **Intracellular expression of α-synuclein must be available to feed the aggregate pool but may not always scale linearly with Lewy-related pathologies**. The availability of α-synuclein monomers as substrates for inclusion formation will depend on a balance between α-synuclein transcription/translation rates, clearance, and multimer formation and aggregation in both patients with Lewy body disease and experimental models thereof. *SNCA* expression in humans varies considerably across tissue types and brain subregions (Fig. 1). In the C57 mouse that is commonly used to model α-synucleinopathies, the *Snca* gene is also not uniformly expressed across brain subregions (Fig. 2). For example, subcortical structures display less *Snca* mRNA, with exception of the dorsomotor nucleus of the vagus and the substantia nigra, pars compacta—both of which are notably vulnerable to Lewy pathology in humans (arrows in Fig. 2).

**Fig. 3:**
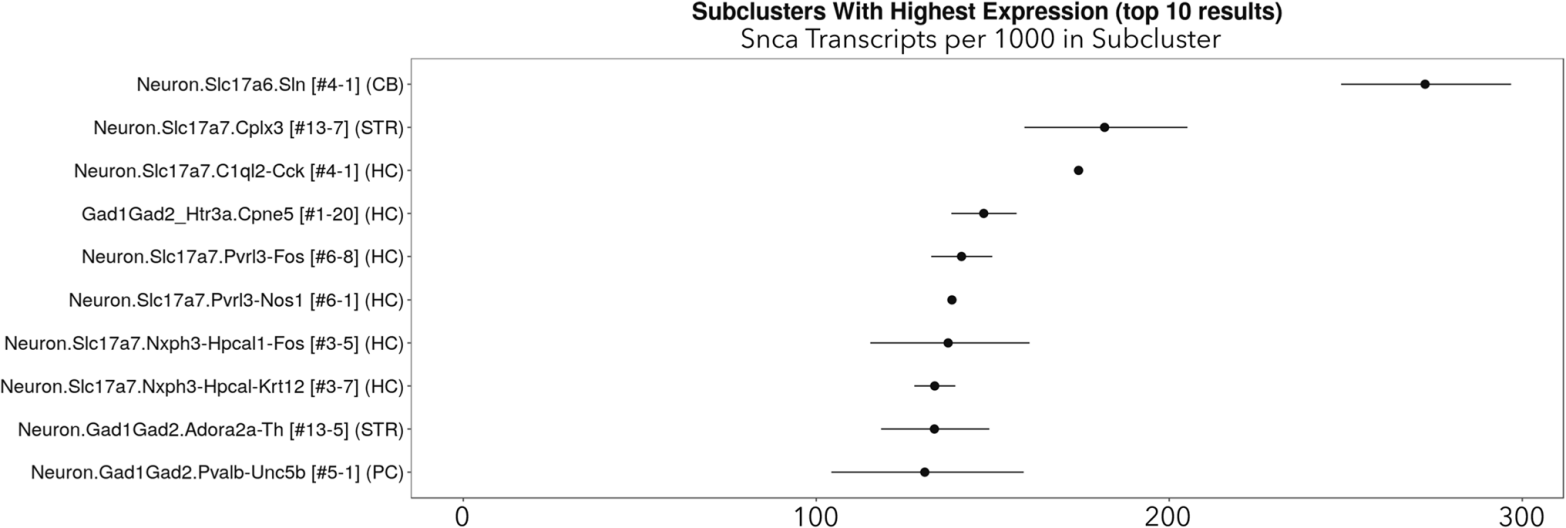
DropViz database ranking of top ten mouse brain cell clusters with the highest expression of *Snca* [207]. Note the high expression of *Snca* in cerebellar neurons and in hippocampal neurons, consistent with mouse data in Fig. 2d. Retrieved from http://dropviz.org
  Other heterogeneous brain regions, such as the hippocampus, are also not major flashpoints for somal Lewy pathology (i.e., Lewy bodies) in humans, and yet express abundant α-synuclein mRNA in both humans (Fig. 1) and mice (Fig. 2 and Fig. 3; also see [112]). Thus, intracellular α-synuclein concentration is only one of the drivers of hippocampal Lewy pathology, alongside the existence of neuronal connections as roadways for spatial transmission [52, 149, 163, 175, 195]. On the other hand, characteristic Lewy-like structures and cell loss fail to develop in experimental models after knockout of *Snca* [120, 200, 258, 260], which has two important implications. First, the presence of α-synuclein is a prerequisite for formation of pathologic inclusions in mice. Second, preformed fibrils per se do not disrupt membranes and other cell components sufficiently to cause frank cell loss in the absence of α-synuclein. It would be helpful to evaluate Lewy-related pathologies in *Snca* knockout mice with tools not inherently dependent on α-synuclein expression. If the gene is absent, so will be the protein, and it will therefore be useful to test, at both microscopic and ultrastructural (cryo-electron microscopic) levels, if filamentous, eosinophilic, and lipid-filled inclusions can form in the absence of the gene encoding α-synuclein.
2. **α-Synuclein is present in both cytosolic and interstitial compartments.** The majority of α-synuclein substrate in the central nervous system is likely to be synthesized inside neurons, but α-synuclein is also released into the interstitial matrix and is therefore present in the cerebrospinal fluid compartment, including in the oligomeric form [64, 65, 153, 184]. Both intracellular and extracellular α-synuclein molecules, including those found inside nanosized extracellular vesicles, may serve as templates for further aggregate formation [92, 253]. Thus, emerging evidence suggests that α-synuclein, including aggregated species of high molecular mass, can be packaged into extracellular vesicles, secreted in a calcium-dependent manner, and transmit pathology to recipient cells [66, 90, 93, 95, 127, 133, 238]. Conditions that encourage extracellular vesicle release may influence the spread of α-synucleinopathy across the neuraxis and open a path for potential entry of α-synuclein aggregates across the blood–brain, gut-to-plasma, and nose-to-brain barriers.
3. **The extracellular aggregate pool requires access to plasma membranes for passage into the cytosol and cell-to-cell propagation**. The probability of uptake of α-synuclein aggregates by a particular neuron may be influenced by how often the neuron samples the extracellular environment by micro- or macropinocytosis, receptors and transporters housed in its plasma membrane, and the binding of cell surface heparan sulfate glycans [103, 108]. One might expect neuronal uptake and release of aggregates to be highest at the synaptic membrane, especially of neurons with extensive synaptic arborization, although the synaptic space is somewhat separated from the remaining interstitial fluid by adhesion molecules and membrane proteins. Given that vesicular membrane recycling is accompanied by pinocytosis of surrounding fluids, the electrical activity of synapses may play a crucial role in uptake and release of aggregates [275]. Human dopamine neurons have been estimated to arborize into more than a million synapses each, possibly extending more than four meters in length, which also places a heavy energetic burden on the mitochondrion [192]. If aggregate uptake occurs along the length of the axon, the degree of myelination may also influence entry of aggregates into neurons [31, 32, 34]. The myelin sheath is not only associated with lower energy expenditures and therefore, less leakage of reactive oxygen species from the electron transport chain, but it also endows the axon with a simple physical barrier. Hence, intersubject differences in active neuronal circuity, density of synaptic terminals, plasma membrane molecules that bind/transport α-synuclein, pinocytic rates, membrane surface area, axon length, and the degree of myelin coverage could underlie variations in α-synuclein aggregate engulfment and partly explain the diverse clinical outcomes of Lewy body disease.
4. **Aggregates may be released from damaged or dying cells and attract immune cells by chemotactic means**. If dying cells are not quickly and fully cleared by microglia, astrocytes, or phagocytic peripheral immune cells that infiltrate the brain, aggregated forms of α-synuclein could be released through damaged membranes into the extracellular compartment for further seeding and expansion of the aggregate pool. In this scenario, a robust inflammatory response may not be as toxic as often assumed but may help isolate and destroy α-synuclein aggregates. Therefore, the hunt for and destruction of damaged or dead neurons harboring α-synuclein aggregates and other toxic cargo by immune cells may slow the spread of Lewy-related pathologies, a scenario consistent with the chemoattractant properties of α-synuclein [100, 236, 267]. These speculations are supported by in vivo observations of microglia/macrophage efferocytosis of neuronal somata housing exogenous α-synuclein fibrils (Fig. 4), and raise the possibility that neurodegeneration is not always harmful in the diseased brain. On the other hand, microglia also discharge α-synuclein within extracellular vesicles that encourage the cell-to-cell transmission of pathology, hyperphosphorylation of α-synuclein, and dopaminergic neuron loss [93]. These collective data likely reflect the wide spectrum of immune cell functions.

**Fig. 4:**
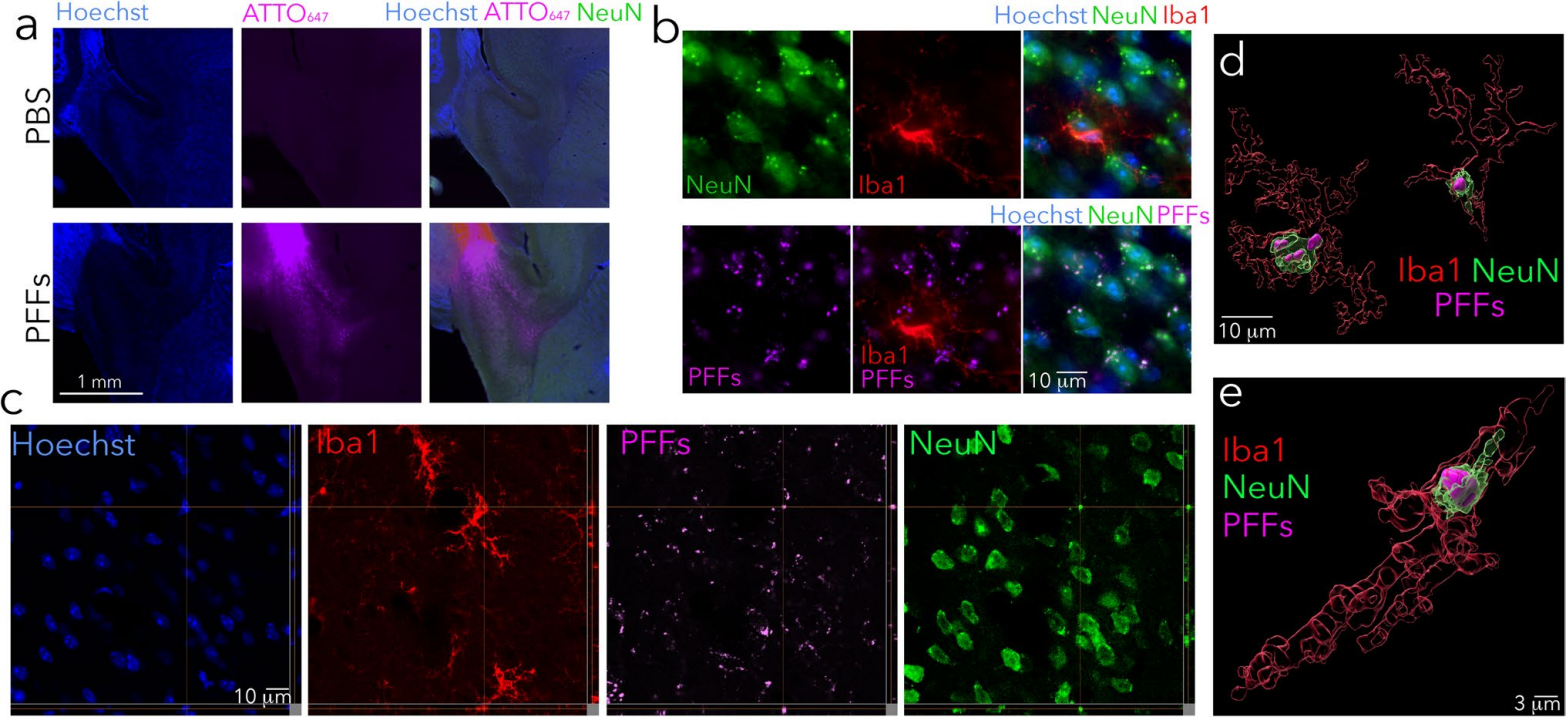
Microglia/macrophages may engulf neurons that house exogenous, preformed fibrils. Phosphate-buffered saline (PBS) or ATTO_647_ preformed fibrils (PFFs) were sonicated and infused in the bulbar anterior olfactory nucleus in genetically outbred CD-1 mice of both sexes, as described [21]. Mice were sacrificed 3 h or 3–6 days later for immunostaining with antibodies against the pan-microglia/macrophage marker Iba1 (Wako 01919741) and the neuronal nuclear marker NeuN (Millipore ABN90). The Hoechst reagent was applied to label all nuclei. **A** Infusion site in the dorsal anterior olfactory nucleus and the caudal olfactory bulb. **B** The labeled markers are shown in isolation (for clarity) or in merged form at three days post-infusion. Note the Iba1^+^ cell partially enfolding a NeuN^+^ cell. Most PFFs at the site of infusion were in NeuN^+^ neurons rather than NeuN^−^ profiles. Thus, we speculate that neurons may be unable to fully degrade aggregates—or else the PFF model would not work. **C** Shown is a confocal Z-slice at the intersection of an Iba1^+^ object engulfing a NeuN^+^ object that houses fluorescent PFFs. **D**–**E** Three-dimensional Imaris rendering of the cell from panel **C**. A second representative example is included on the left of panel **D**
5. **Aggregates are incompletely removed from extracellular and intracellular compartments by the available clearance mechanisms**. The clearance of protein aggregates by autophagic or proteasomal degradation, the sequestration of α-synuclein in mature Lewy bodies, and the extrusion of toxic aggregates into the extracellular matrix for uptake by neighboring glia (or removal by glymphatic clearance) all conceivably impact the course of Lewy body disease but are not adequate to fully contain the spread of protein aggregates. Undigested aggregates can rupture lysosomal membranes, leading to autophagic failure, unless lysophagic clearance systems can be fully engaged [115]. Each of these defensive systems will closely depend on the metabolic and energetic resources available to the cell for repair and recovery. Other defenses against protein aggregates include tunneling nanotubes across glial cells, perhaps as a means of diluting the toxicity and sharing the burden of degrading α-synuclein aggregates with other cells by autophagy [48, 211]. However, high concentrations of α-synuclein may saturate microglial autophagic systems [248]. It seems worth cautioning that the presence of α-synuclein mRNA and protein within glial and other non-neuronal cells does not necessarily imply that these cells transcribe *Snca* mRNA from nuclear DNA. Rather, single-cell transcriptomic sequencing and flow cytometric analyses of single cells may also detect mRNAs and proteins engulfed from non-autonomous sources that are not yet degraded, including via uptake of extracellular vesicles or glial efferocytosis of neurons containing α-synuclein (Fig. 4).
6. **The properties of extracellular matrices influence aggregate diffusion**. There are few studies of the extracellular compartment in Lewy body disease, but α-synucleinopathy has been demonstrated to alter the structure and function of extracellular matrix components, including forcing the degradation of hyaluronan [229]. The flow of protein aggregates alongside neuronal membranes may be influenced by adhesion to components of the extracellular matrix (including lipophilic components), binding to plasma membrane proteins and carbohydrates that protrude deep into the interstitial fluid, repulsion from extracellular water, and the flow and shear dynamics of the extracellular compartment [111, 169, 229].
7. **Aggregates accumulate with age and/or the passage of time**. In several models of Lewy body disease, older animals tend to develop more pathology than younger animals [42, 105, 121, 254]. Advanced age is the chief risk factor for Lewy body disease, perhaps due to loss of protein quality control with aging and accumulation of undigested protein aggregates, following cumulative lifetime exposures to environmental and intrinsic insults (e.g., reactive oxygen species from oxidative phosphorylation). In the absence of a degenerative disorder, aging is not accompanied by robust, nonspecific neuronal loss [261]. However, aging may be accompanied by a decay of immune cell function [272], which could permit the escape of α-synuclein aggregates from one cell into another (see above). Another major impact of aging and the passage of time is the amassment of DNA damage [215], particularly in postmitotic neurons that cannot divide and that escape repair mechanisms, such as base-excision repair. This accruing burden might lead to diversion of energetic resources and structural changes in proteins translated from damaged sequences in nuclear or mitochondrial genomes. Over time, age-related proteomic shifts may surpass the threshold for a functional neuronal or glial cell, leading to age-related neurological deficiencies and engagement of cellular senescence programs.

## Conclusions

Research on α-synuclein has progressed to the point that patient-tailored anti-α-synuclein therapies may be on the horizon. Clinicians can measure α-synuclein aggregation propensities through α-synuclein seed amplification assays conducted on patients’ cerebrospinal fluid, blood, or biopsies of solid tissues where Lewy-related pathologies tend to form [40, 123, 155, 180, 222]. The mechanism of the α-synuclein seed amplification assay is easy to envisage, because filamentous structures tend to naturally get nested together with vigorous shaking in a confined box, and the rate of alignment is accelerated the more filaments are aligned at the beginning of the reaction. As the structural aspects of the filaments (e.g., smooth or bumpy) will influence the kinetics of such reactions, one can also envision that diverse spatial qualities of α-synuclein strains influence clinical phenotypes and rates of disease progression [129, 162]. The contribution of polymorphic strains of α-synuclein filaments to disease progression is particularly evident when comparing Lewy body disorders to multiple system atrophy, an α-synucleinopathic condition that is not classified as a Lewy body disorder [102, 119, 216, 228].

Although spatial patterns of Lewy inclusions are not uniformly correlated with neurodegeneration (e.g., see discussions by [241] versus [14]), the α-synuclein seeding capacities of antemortem cerebrospinal fluid samples appear to correlate with postmortem evaluations of the presence and anatomical reach of Lewy inclusions in the brain, suggesting that the number (and shapes) of pathogenic seeds are associated at least to some degree with the transmission of Lewy-related pathology across the neuraxis [7, 18, 205]. Because these tests can be carried out on live subjects, they may be useful to stratify patients prior to clinical trials as α-synuclein-targeted therapies become available [230]. For example, patients with parkinsonism but without positive α-synuclein seed amplification may be inappropriate to include in clinical trials of monoclonal antibodies against α-synuclein.

In conclusion, aside from processes listed above, α-synuclein aggregates may form, spread across cells, exert toxicity, and induce cell loss depending upon the following factors:

- The number of α-synuclein molecules that crowd the cell and are available as nucleation points for additional seeding and phase separation
- The intracellular location of α-synuclein aggregates (e.g., possibly more toxic or disruptive if in a thin axon or small synapse, rather than tucked within Lewy bodies in a sequestered corner of the roomier somata)
- The compactness/maturity/insolubility of the inclusion and its interactions at inclusion boundaries with cytoskeletal proteins and mitochondria, lysosomes, and proteasomes
- The tendencies of noncompact inclusions to break apart and leach soluble oligomers

There are many other factors we have not considered here. Additional research on the biophysical and biochemical underpinnings of Lewy body disease, the expansion of α-synucleinopathic lesions over space and time, and the molecular means of initiation of cell death programs will likely be needed to conduct effective clinical trials and slow or prevent Lewy body disease.

## Data Availability

Data will be made available upon request to leakr@duq.edu.
